# Newly Qualified Canadian Nurses’ Experiences With Digital Health in the Workplace: Comparative Qualitative Analysis

**DOI:** 10.2196/53258

**Published:** 2024-08-19

**Authors:** Manal Kleib, Antonia Arnaert, Lynn M Nagle, Rebecca Sugars, Daniel da Costa

**Affiliations:** 1 Faculty of Nursing University of Alberta Edmonton, AB Canada; 2 Ingram School of Nursing McGill University Montreal, QC Canada; 3 Faculty of Nursing University of New Brunswick Fredericton, NB Canada

**Keywords:** digital health, new graduate nurses, nursing practice, workplace, informatics

## Abstract

**Background:**

Clinical practice settings have increasingly become dependent on the use of digital or eHealth technologies such as electronic health records. It is vitally important to support nurses in adapting to digitalized health care systems; however, little is known about nursing graduates’ experiences as they transition to the workplace.

**Objective:**

This study aims to (1) describe newly qualified nurses’ experiences with digital health in the workplace, and (2) identify strategies that could help support new graduates’ transition and practice with digital health.

**Methods:**

An exploratory descriptive qualitative design was used. A total of 14 nurses from Eastern and Western Canada participated in semistructured interviews and data were analyzed using inductive content analysis.

**Results:**

Three themes were identified: (1) experiences before becoming a registered nurse, (2) experiences upon joining the workplace, and (3) suggestions for bridging the gap in transition to digital health practice. Findings revealed more similarities than differences between participants with respect to gaps in digital health education, technology-related challenges, and their influence on nursing practice.

**Conclusions:**

Digital health is the foundation of contemporary health care; therefore, comprehensive education during nursing school and throughout professional nursing practice, as well as organizational support and policy, are critical pillars. Health systems investing in digital health technologies must create supportive work environments for nurses to thrive in technologically rich environments and increase their capacity to deliver the digital health future.

## Introduction

Clinical practice settings have increasingly become dependent on the use of digital or eHealth technologies such as electronic health records, telehealth, mobile health, and medical devices to name a few [1,2]. The COVID-19 pandemic has also contributed to the increased use of digital technologies to facilitate virtual care delivery, creating both opportunities and challenges for care providers and patients [3-5]. Digital health refers to the “proper use of technology for improving the health and well-being of people at the individual and population levels” [6]. According to the World Health Organization, digital health expands the concept of eHealth, with a wide range of smart devices and connected equipment. It also encompasses other uses of digital technologies for health such as the Internet of Things, artificial intelligence (AI), big data, and robotics [7].

Recognizing the increased use of digital health across health systems, the International Council of Nurses [8] has recently released a position statement affirming the central role of digital health in contemporary nursing practice and the importance of developing the skills and competencies of nurses through the integration of digital health content in formal undergraduate and postgraduate curricula and participation in continuing professional development. These recommendations have also recently been echoed by the Canadian government [9] identifying digital preparedness as one dimension of actionable strategies that should be put into place to mitigate the administrative burden associated with the use of digital tools by nurses as primary users of these technologies.

Digital capabilities or informatics competencies are critical core requirements for safe nursing practice with technology. In Canadian health care, nurses must be able to use digital health technologies to support information synthesis and patient care in accordance with their professional and regulatory standards [10]. Despite efforts to enhance digital preparedness among future nurses, research involving senior-level nursing students identified that most learning about digital health is taking place in clinical settings with limited theoretical education in the classroom [11,12]. In a practice-based profession, such as nursing, it is inherent that nursing students are socialized into their professional roles by observing and interacting with nurses [13] in health care settings that they go to for their clinical education; however, practicing nurses involved in mentoring nursing students also have limited digital capabilities and report challenges with technology use, mostly among older nurses [14,15]. Assumptions also prevail that younger nurses are tech-savvy because they were mostly born in the digital age; however, despite being described as digital natives, research suggests that they do not naturally have positive views on using technology for care provision [16] nor do their digital skills easily transfer to the clinical context [13,17-19].

While nursing programs acknowledge the importance of integrating digital health content at all levels of nursing education, research involving nurse educators [20] and academic administrators [21] from across schools of nursing in Canada revealed that nurse educators have limited knowledge and capacity to teach informatics, limited awareness about informatics competencies, and limited resources to teach nursing students about digital health applications in clinical practice. For example, 65% of these schools indicated they do not have access to a training version of any electronic health record system to teach students about this technology. Although these observations are focused on Canadian nurses, similar gaps have been reported in other countries [18,19,22,23]. Potentially, these gaps can have a negative impact on nursing graduates as they transition to the workplace. According to a recent study [24] involving clinical managers and newly registered nurses (RNs) in the United Kingdom, researchers identified several factors impacting these nurses in the workplace including technology infrastructure, time, skills, digital literacy training, support, leadership, familiarity, and confidence: creating barriers to optimal nursing practice with technology. It is vitally important to support nurses in adapting to more digitalized health care systems; however, little is known about Canadian nursing graduates’ experiences. This study sought to (1) describe newly qualified nurses’ experiences with digital health in the workplace, and (2) identify strategies that could help support their transition and clinical practice with digital health technologies.

## Methods

### Design

An exploratory descriptive qualitative design was most appropriate considering the limited research available [25]. This design aligns with the philosophical perspectives of pragmatism and constructionism and is concerned with understanding and describing the human experience within its unique context [25]. Through inductive and dynamic research processes, the researcher explores the subjective experiences of participants and their perceptions through a collection of data that describes the “who, what, and where of the events or experiences” [25]. In this study, qualitative data was collected from semistructured interviews with nurses, a technique suitable for collecting data that is open-ended and explores participants’ thoughts, feelings, and beliefs about a particular topic [26].

### Participants

Qualitative studies recommend 1-30 informants [25-28]. In this study, the number of participants was guided by the concept of information power to interview sampling, which indicates that if the sample holds more information that is relevant to the actual study, a lower number of participants is needed [27]. The five aspects of information power further supporting this approach were (1) the specific or narrow study aim, (2) participants holding characteristics that are specific to the study aim and identification supported by suitable recruitment strategies, (3) theoretical support, (4) the quality of dialogue with participants, and (5) a thematic cross-case analysis approach [27]. In addition, the concept of thematic saturation, which means researchers would stop data collection when there is no new information shared by participants [26], was also considered. Participants were newly qualified nurses who graduated from 2 undergraduate nursing programs in Eastern and Western Canada (WC) in the last 2 years and have been working in clinical settings that have digital health technologies, such as electronic health records, regardless of the stage of implementation. Purposive and snowball sampling techniques were applied to enroll interested participants with consideration of various sociodemographic backgrounds [28].

### Data Collection

To recruit participants, administrative staff in the selected nursing programs circulated a recruitment poster invitation via a listserv email to graduates from these programs, and potential participants were asked to contact the researchers to express interest in participating in the research. An interview guide (Textbox 1) was developed by the researchers based on their expertise in this area and the general literature using open-ended questions and prompts to facilitate the discussion regarding aspects related to new graduates’ experiences with digital health [26]. Pretesting of the interview schedule was accomplished by engaging 2 research assistants (RAs; RS and DC) involved in this project in a demo interview, as both were recent graduates. No further revisions were introduced to the initial interview guide. In addition, sociodemographic data, including age, gender, and work setting, were collected at the beginning of interviews to understand the characteristics of participants and the context of their practice environments. Interviews were facilitated by RAs (RS and DC) without the presence of the researchers who were also faculty members and may have interacted with these participants when they were students, as such minimize undue discomfort to participants. The interviews were held via Zoom (Zoom Video Communications, Inc), each lasting between 45 and 60 minutes, and participants had the option to turn their cameras off during the interview. Although options for in-person interviews were offered, participants preferred a digital approach considering their busy clinical schedules, and this mode did not impact the quality of dialogue between the participants and the interviewers. The audio-recorded interviews were then uploaded to a secured SharePoint (Microsoft Corp) drive and later transcribed verbatim by a professional transcriptionist service provider and records.

**Interview guide.**

- Could you share what types of technologies do you currently use in your day-to-day practice as a registered nurse? Prompt: Could you share some examples?
- Could you describe your experiences when working with these technologies? Prompt: what makes your work easy/difficult?
- Could you describe your experiences of learning on how to use these technologies? Prompt: In the nursing school/in the workplace?
- What suggestions do you have to enhance newly graduated nurses’ experiences of using digital health technologies? Prompt: in the nursing school, in the workplace, other?
- Is there anything else you would like to share?

### Data Analysis

RAs (RS and DC) received training in qualitative data analysis and an orientation to the data management software. Data were uploaded into the NVivo software (Lumivero) in a deidentified format to facilitate data management and analysis. An inductive manifest content analysis was used considering the research is exploratory. In this approach, the goal is to “describe what the informants actually say, stay close to the text, use the words themselves, and describe the visible and obvious in the text” [28]. The unit of analysis (ie, the sample) involved analyzing data from all the participants in its entirety [28]. The two RAs were involved in data analysis, which began by having each one independently read the interview transcripts line by line, several times, to make sense of the data and whole and achieve familiarity [28-30] Open coding (ie, identification of meaning units and labeling each with a code) was then done by having each RA independently analyze 2 transcripts. These pilot transcripts were then compared and discussed to enhance reliability before coding the remainder of the data. At this stage, we also created a coding list, including all the meaning units identified along with an explanation of the codes to enhance intercoder reliability and reduce cognitive fatigue. The initial coding was then done by the research team without additional changes introduced. Condensing of the meaning units was then done to identify categories and subcategories without losing the content of the unit, which was followed by grouping of categories with similar events into main categories. The researchers then constructed the themes based on the analysis of these data (Multimedia Appendix 1). The resulting analysis was reported in a narrative format. Rigor was enhanced by reviewing the coding scheme against the interview data and the involvement of all team members in the discussion and interpretation of the findings [28-30].

### Ethical Considerations

Ethics approval was obtained from an Ethics Research Review Board in the WC research site (Pro00112596), and secondary approval from an ethics board at the university in Eastern Canada (EC; A12-E69-21B). Participants were provided with an information sheet along with the consent form and were given the opportunity to ask questions and verify any aspect related to the research before providing their consent for participation. Participants were also assured that they could opt out at any time without penalty. To protect privacy and confidentiality, the interview data were deidentified by using a code (eg, participant 01-E and participant 01-W). Each participant received a small gift certificate to convey appreciation of their time.

## Results

### Demographic Characteristics of Participants

A total of 14 participants were interviewed, 6 participants from WC and 8 participants from EC. Of those 14 participants, 8 participants were younger than 25 years old, 4 participants were in the age category of 26-30 years, and 2 participants were in the age category of 31-41 years. Participants were mostly females, except for 1 male participant. They worked in large urban hospitals in a variety of units including emergency room, intensive care, internal medicine, trauma surgery, and operating room.

### Digital Health Technologies in the Workplace

In their current practice environments as RNs, participants indicated that they used different hardware (eg, laptops, mobile devices, workstations on wheels, and portable computers), clinical information systems (CIS), and medical devices and equipment (Table 1).

**Table 1:** Types of digital health technologies currently used in practice.

Technology	Western Canada site	Eastern Canada site
CIS^a^: new, old, and hybrid systems	EPIC CIS/EMR^b^ EDIS or Millennium NetCare Electronic Health Record Pyxis Vax Meditech	OACIS (EMR used universally) MedUrge (EMR used in acute care only) VSign (mobile app to access Oacis remotely) PANDAWebRx (formerly known as GESPHARxLite; eMAR used at some sites)
Medical devices and equipment (some devices are connected to newer CIS systems, and others are stand-alone with paper charting)	Sphygmomanometer for blood pressure Cardiac monitors Glucometers Bladder scanners Transcutaneous bilirubin meter Dopplers ECG^c^ machines Pulse oximeter for O2 Saturation Temperature probe	Philips monitors (syncs to Oacis) Nova glucometers (syncs to Oacis) BBraun IV pumps Apple watches (in terms of patient education and telemonitoring) Continuous glucose monitors used by patients

^a^CIS: clinical information system.

^b^EMR: electronic medical record.

^c^ECG: electrocardiogram.

### Key Findings

#### Overview

Data analysis revealed three themes: (1) experiences before becoming an RN, (2) experiences upon joining the workplace, and (3) suggestions for bridging the gap in the transition to digital health practice. These themes are discussed below with illustrative quotes from the participants.

#### Theme 1: Experiences Before Becoming an RN

This theme includes the categories: (1) academic, (2) clinical, and (3) personal experiences. Most participants stated having familiarity with technology before joining their schools, and perhaps because of that, it was assumed that they were highly literate with technology since they grew up with it and have used it frequently in their daily lives. In a way, this made learning about technology easier, but in other cases, it also created barriers. According to one participant:

...sometimes I feel like there’s an assumption that maybe we know more than we do, just because of the age we are ... we know how to use things like a phone, or the computer, or whatever, but it’s used in a different way in practice.

P5, WC

This sentiment was shared by another participant who noted that individual differences should also be considered:

I think it’s individual, some people are just a little bit more inclined to be using it [technology] and have an easier time.

P2, EC

At the time participants were in school, they indicated being aware of the relevance of digital health technology (DHT) to nursing practice. Their education involved varied exposure to theoretical and laboratory training, with most learning taking place in clinical settings, but this was not without challenges. A participant described it as follows:

It was very much like somebody handed you a cheat sheet that just told you like, Type in these numbers, and then you will find your [Laughs] ... your medication page for this patient, and you can enter your medications. So, we got that from our clinical instructor, and then different nurses had different workflows. They would show us, this is how to print out my MAR, or this is the time of day when I’ll go and log that I’ve given my medications.

P1, WC

The depth and breadth of these experiences were also noted by another participant who further elaborated:

At the beginning of every clinical, we had somewhat of an orientation on the first day where we would go into the unit where we’d be doing our stage and we would get a little session on how to use the different platforms that the unit uses ... They would show us a brief overview of the different tabs that are available and what kind of information you can get and that kind of stuff.

P3, EC

Another participant added:

Even just things of like having to do an ECG on someone ... Where do I put the other ECG tab? Because that’s an actual thing that happens ... nobody comes in looking perfectly healthy and pristine as like the students that are in the program.

P7, WC

With respect to learning experiences in the classroom, the majority indicated receiving limited and broad education about DHT, with some having mixed feelings about these gaps. As described by one participant:

We would talk about it in lectures ... I don’t really remember ever doing anything like digital in labs.

P5, WC

Another participant agreed adding:

During my studies, it was less obvious ... it wasn’t through my classes that I learned about these technologies, it was more when I was using it in practice.

P7, EC

One participant went on saying:

There are so many unspoken things in nursing ... I always found myself, a lot of times, getting feedback from the instructor saying, “You should know this,” and me being like, “I didn’t know that I had to ... I didn’t know that I didn’t know that I was supposed to know this.

P7, WC

Some participants did not make sense of DHT and its relevance to nursing practice until they had experienced its expanded use; somewhat creating a sense of urgency that they should learn more about it. A participant explained:

It wasn’t until the pandemic, really, that I understood. Now, this is what telehealth is used for; this is the function, this is what it can do, it can bridge the gaps ... it was mostly the pandemic that really changed my mindset there.

P8, EC

The large-scale transition to a new electronic record system was also mentioned:

...like not built into our curriculum, for example, when we would have guest speakers or informal discussions, we would always talk about electronic charting system because it was like a pretty big deal for the implementation of this provincial system in phases...

P4: WC

#### Theme 2: Experiences Upon Joining the Workplace

This theme includes two categories: (1) facilitating and (2) hindering experiences regarding the use of DHT in the workplace.

Hindering experiences discussed by participants included aspects related to training on CIS and medical devices and support resources, the IT infrastructure, and the potential impact of these challenges on their practice. Upon starting their practice as novice nurses, most participants indicated receiving either limited or condensed DHT training over a short period, which despite their tech-savviness, found to be a little overwhelming. This thought was shared by a participant who mentioned:

Our orientation for [__] was fairly short ... it was a lot of self-learning on how the program works ... you just have to play around with the program to kind of understand how it works.

P1, EC

Another participant added:

We never received any kind of formal training on how to interact with patients using telehealth. Not even in formal training, you are kind of thrown into that world and expected to pick up the phone and have these conversations.

P3, EC

Another participant shared

They’ll teach us if we’re the nurse for that patient on how to use it [a medical device]. But it’s not like a full unit thing ... it’s kind of limited for 30 minutes ... and then the one nurse that has the patient that day. I mean, when that nurse leaves, another nurse comes in, how are they going to help the patient with this device?

P1, EC

Participants also mentioned fragmentation of digital health across the health care system, exacerbated by a lack of interoperability, with less technology integration in rural settings. When working with hybrid or outdated systems, new graduates felt more prone to making errors while transferring patient information between different platforms. As one participant described:

I’m not looking to make my job easier. I’m looking to make myself more effective, and I would be significantly more effective if I had access to everything and could spend less time fumbling through papers and, you know, clicking on screens, and could do things faster, then, I can spend more time actually with my patients...

P1, WC

Participants also discussed that patient care became less efficient when the system was not user-friendly, the technology was outdated, the system lagged, IT glitches were not solved quickly, and there were not enough computers on the unit. All these issues increased concerns regarding patient privacy and confidentiality, safety, and potential legal liabilities to the nurse and the organization. A participant explained:

Because sometimes that happens, you know, one computer at the nursing station is not working, you go on another one, everything is down. And it came back within like 15-20 minutes. That’s a long time when I need to call a doctor to see a critical value. Maybe it doesn’t sound like a lot, but 15 minutes for the system, the entire system to be down is a lot. And when that was going to happen, it was a near change of shift. So, we weren’t sure if the care plans were going to print for the next shift. So, we had to start writing them out. This is not a feasible way to be doing this. In that case, we were all lost. Even the people who had been there for 20 years didn’t know what to do. I was asking people, “What can I do here?” Nobody knew. So, not really good.

P8, EC

These practices were perceived to consume valuable nursing time that could have been spent in the clinical care of patients as a participant explained:

When we take time trying to fix a machine that wasn’t working so that we could take patients to monitor their cardiac function, then it takes away from our ability to get to know patients and to treat them more holistically.

P5, EC

Multiple participants also voiced concerns regarding inconsistencies in how nurses documented electronically, and how these differences although sometimes helped them learn different strategies, also caused confusion and could potentially result in legal or patient safety issues as one participant described:

I think there are some discrepancies that could lead to some actual challenges for patient safety.

P5, EC

Some also indicated redundant charting or copying electronic information from one platform to another, sometimes including an analog step requiring a physical document to be scanned. According to one participant:

Well, I know something happened that day, but nothing got charted about it, or there is not very complete charting about that incident, because that nurse prefers to chart everything in her flowsheets and doesn’t write a lot in her note.

P2, WC

These issues were further compounded by the limited support and resources available to new graduates during night shifts and on weekends such as access to clinical nurse educators (CNEs) and tech support as one participant explained:

When you are on a dayshift, there’s lots of extra support. It is a little bit tougher if you are on evenings or nights because those extra supports, like the managers and educators, aren’t around.

P2, WC

On the other hand, participants discussed factors that facilitated their experiences with DHT including having access to a wide range of technologies to support care, being adaptable, and positive workplace culture. Having access to technology that worked well enhanced efficiency including time-saving actions such as the convenience of having prompt access to patient information in one system, which enabled prioritization of care, clinical decision-making, standardization, and continuity of care. According to one participant:

I think it [CIS] has definitely made me more detail-oriented, especially now that I’ve been using them for a little bit over a year ... there are so many things that you’re thinking about day-to-day with every single patient, ... okay, what are the priorities for my patient, and what am I looking for? What could go wrong? What do I need to keep in mind?

P2, WC

Some participants also talked about the technology being helpful in giving them prompts about things they have learned about but could not easily remember. For instance, some of the newer CIS systems provided help pages with information about managing patient care and relevant nursing actions, which facilitated their practice in busy clinical environments as they were also adapting to their new roles as independent RNs:

It’s been a great facilitator in my learning experience as a new grad nurse because I’m able to stay on top of everything more easily ... it gives us the opportunity to focus on patient interaction and on what's important.

P5, EC

Participants acknowledged that learning about technology is a learning curve and therefore being adaptable is key. In addition, ongoing learning about technology is important—not only upon orientation to the unit. As explained by participant:

...there was really just like a different learning curve ... now it’s been like over two years since the system has been implemented, everyone is pretty much onboard ... there’s still some of our staff that, you know, like need help with certain things, every now and then, something will surprise you and like you’ll realize you’re still learning, or like they’ll update the software.

P4, WC

New graduates’ practice with DHT became easier through the support of nurses in the unit, accessibility to other resource persons, such as CNEs and superusers, having the opportunity to learn from more experienced nurses and in exchange teaching them technology-related skills, and the mentoring behaviors of nurses toward the new hires. According to one participant:

The culture of the unit is so supportive in so many ways, I think that it exceeded all my expectations for the support that I feel in being able to ask questions and share knowledge with other people.

P5, EC

Other factors contributing to positive experiences, as discussed by some participants who have transitioned to a new system, included working with integrated CIS systems and having access to a playground or a sandbox during their initial training. In addition, those who have had positive training and education experiences with DHT as nursing students were also more likely to support new incoming nurses as they transitioned to their new workplace as described by one participant:

Because I was a student when I started using [___] in practice, I got a little bit more of a transition into how the system worked ... when we’ve new hires come in who’ve never used electronic charting before, we always try to go around and see patients together, and we’ll say like, “Okay, you’ll chart with so-and-so today, so that, you know, you can get used to the system and play around with it, and just get comfortable.”

P4, WC

#### Theme 3: Suggestions for Bridging Gaps in Transitioning to Digital Health Practice

Participants shared different strategies on how to improve newly qualified nurses’ practice with DHT discussing aspects related to improving the education of nursing students, and strategies that can be introduced to enhance new graduate nurses’ experiences once they join the workplace.

With respect to nursing education and looking back at their own experiences as nursing students and recognizing the expanded use of technology in the health care system, participants would have appreciated if they had had adequate education and exposure to DHT during their school to help them make better sense of how DH relates to nursing practice and the clinical care of patients. One participant shared:

I feel like a lot of nursing school time was spent on different things that don’t necessarily translate to current practice, nor does it translate to digital health information. I’ve never made a digital care plan in my time as a registered nurse, but we did spend a lot of time on care plans in school.

P3, WC

They felt nursing education could be improved by providing relevant hands-on learning that goes beyond knowing how to use the system to critically think about why a nurse is doing these actions. The participant went on to share an example of how this looks like to them:

...Here’s this patient that you have. These are the computer systems that you’re using. What are the checks that you need to do on the computer system? What do you need to look at? What information do you need to pull out of the system? How would you chart for this specific patient? “How do you interact with other disciplines within the specific computer system? ... Like that would have been very helpful.

P3, WC

Recognizing the changes taking place in health care with respect to technology integration, participants stressed the importance of having both dedicated and focused theoretical and clinical education on core concepts relevant to DH in undergraduate nursing education. Examples of the topics that participants discussed included the “how” and “why” of DH technology, ethical issues involving the use of DH technology, privacy, confidentiality, information security, legal implications, medical devices, and the different DHTs currently in use in hospitals and community settings, as well as newer innovations, technologies, and services that will be introduced in the future. According to one participant:

The reality is that tech is really taking over, and it is the future. I’m hoping that manually writing in charts is going to be a thing of the past. And so, to integrate that as early on as possible, the better, it’s a reality, it’s there. There’s no need to leave it on the sidebar until later.

P2, EC

Another participant mentioned:

I think that having a whole course on digital health would be very interesting, because I feel the topic of artificial intelligence is a big topic, especially recently, and there’s a lot of innovation happening with that ... I think it would need to be already applied in the hospitals though for them to fully absorb the information and see the practical application of what they’re learning.

P1, EC

Participants also emphasized the importance of exposing nursing students to DHT used in clinical practice; creating opportunities for hands-on learning while in the nursing program as opposed to learning about them when opportunities arise in clinical training sites. These experiential learning experiences were viewed as essential to enhance confidence and competence. One participant shared:

Definitely, built-in training in school for everyone, not just if you’re going to a hospital that uses an EMR. I think everybody needs it.

P4, WC

Another participant elaborated:

I think having maybe a seminar on how to use particular systems, such as MEDITECH, VAX, if these are systems that are widely used in hospitals, and then in your clinical settings, it’d be nice to kind of have that training beforehand, even just like an introduction to these types of things. Because at school, the training was mostly on the unit, for that specific clinical course.

P3, WC

Regarding workplace strategies, participants emphasized the need for creating opportunities for nurses’ engagement, particularly younger ones, in technology-related decisions and the role of nursing leadership in this process as one participant explained:

It seems like a lot of time, there’s not a younger generation involved in the process [technology] ... they’re much led by people with experience.

P7, WC

Noting the expansive digitalization taking place in health care, another participant added:

I definitely think that there should be safety checks if you’re really going to be pushing everything into Digital Health, everything needs to be looked after and evaluated or tested properly before it’s implemented ... I think you can evaluate it from different ways, like patient outcomes, you could look at the nursing team, the people who are using it day-to-day, you can evaluate them, you can see where they’re at with the use.

P8, EC

Participants also emphasized the importance of creating opportunities for professional development and ongoing learning as the field of technology continues to evolve:

Continuous learning ... that’s something that will always be necessary when it comes to technology, based on the advancements that are made every day and hopefully will continue to be made within these units.

P5, EC

These strategies were viewed by participants as an indication of support and actions that convey commitment from the organizations and leaders toward enhancing new graduates’ abilities to effectively adapt to the current technology-rich work environment and make full use of DHT to improve practice and patient outcomes. A participant described:

...that support looks like booking you in for paid training, which the managers do at the site that I’m in. Being advocates for change, and being advocates for a new system, answering concerns that people may have, and having an in-depth knowledge of the system that’s coming, and not just, “Oh, this new system’s rolling out, and other people will know how to use it.

P3, WC

## Discussion

### Principal Findings

This study explored newly qualified nurses’ experiences with digital health in the workplace. Results identified that participants’ experiences in this study were influenced by inadequate education about digital health while in their nursing school in addition to challenges they faced upon joining the workplace including variable training and support, as well as technology infrastructure. Nonetheless, participants also identified several factors that facilitated their transition including having access to a wide range of technologies to support care, being adaptable, and positive workplace culture. Inadequate theoretical and clinical education about nursing informatics and digital health during nursing school creates unnecessary challenges for the already overwhelmed newly qualified nurse [5,23]. These nurses not only have to adjust to the complex practice setting and the team culture but also learn how to use the technology independently while providing care. To some of the participants, their abilities to connect the dots and understand what digital health means were due to events, such as technology implementation or the COVID-19 pandemic, which also made them further question their readiness for digital health.

Educating nurses about digital health and nursing informatics is an area that has received much attention in the literature. Prior research has shown that educators and nursing programs are often challenged and confused about what informatics means, how it relates to nursing practice, and the application of digital health in care delivery [31,32]. In part, this may be attributed to the availability of multiple informatics competency lists; each presenting different perspectives on what to teach students and these are also getting dated with no focus on emerging technologies such as AI [33,34]. Other research has identified an urgent need for explicit and consistent teaching about digital health (existing and emerging technologies) concepts and their integration into clinical care [18,35,36].

Although there are efforts to bridge the theory-practice gap in relation to digital health education, clinical application experiences remain inconsistent and largely influenced by the availability of technology infrastructure in the schools of nursing and the clinical placement settings [11,13,18,20,21,23,34]. Additionally, limited or lacking professional and regulatory standards to inform nursing practice in digital health is also likely contributing to nurses’ limited engagement with digital health beyond what is required in a specific work setting. These patterns are further reinforced by a persistent model of basic and condensed computer proficiency training programs in the workplace, limited on-site support and opportunities for continued learning, and vague organizational policies; collectively promoting a task-based approach to working with DHT. While the pandemic has served as a catalyst for more engagement in digital health overall, it also exposed several challenges as a result of abruptly transitioning to virtual care delivery with little support and training for care providers on the technologies used and the changing context of practice [5,37]. These factors raise concerns regarding nurses’ capacity to actively participate in the evolving digital health ecosystem continuously being revolutionized by more sophisticated technologies such as AI [20,36-39]; further underscoring the importance of integrated and systematic strategies to address digital health readiness in nursing.

Despite these challenges, in this study, new graduates appreciated the benefits that DHT could provide to improve their practice and patient care. Furthermore, the positive role models and the supportive unit culture enabled a smoother transition, especially for those who experienced a system-wide technology implementation. In that case, senior nurses and newly qualified nurses found themselves in the same boat, and they had to uplift each other into the process. In exchange, new graduates offered to share their digital skills to support more senior nurses who are not as comfortable with technology; thereby, creating reciprocal benefits. Although these strategies proved useful for participants in this study and these behaviors should be encouraged, organizational support should be in place to facilitate nurses’ work with DHT [8,24,40].

Newly qualified nurses in this study clearly recognized that technology integration in health care is on the rise and will likely continue to shape their practice and the practice of future nurses joining the profession in the years to come. Therefore, they emphasized the importance of continuing education, mentorship by nurse leaders, investment in quality CIS, ongoing support from health care organizations, and opportunities for nurses to be more engaged in the design, implementation, and evaluation of current and future DHT as paramount strategies to ensure that nurses are better prepared to effectively lead the digital health transformation. They also emphasized the importance of formal education about DH with meaningful linkage to clinical practice applications.

Academic and clinical learning experiences about digital health are intertwined and contribute to shaping nurses’ perspectives about their practice in digital health. In educating the next generation of nurses, prelicensure nursing programs should provide early, comprehensive, and focused education about DH to enable nursing students to understand the full spectrum of digital health so they are work-ready upon joining the practice setting as independent practitioners [11,17,18,23,39,41,42]. Such education should also include core concepts of digital health, technological advancements and innovation, the benefits and potential impact of these technologies on the quality and safety of patient care, and professional nursing roles [36,37,39] with clear linkages to informatics competency requirements. This could be achieved by providing relevant theoretical content in the classroom and enriching that with experiential learning experiences in the laboratory, simulation, and clinical practicum throughout the program, as these experiences have been found to be beneficial for bridging the theory-practice gap, specifically in the context of learning about main DH technologies such as electronic records [12,22,39].

To improve nurses’ practice in digital health, nursing organizations must also develop clearer and more consistent policies regarding existing and emerging digital health technologies and how these fit within nurses’ scope of practice according to their professional roles [8,39]. Consequently, this translates to clearer digital health expectations for schools of nursing, nursing students, and employers [36,37,39,41]. In the workplace, as opposed to proficiency-based training upon onboarding and orientation to clinical practice settings, newly qualified nurses would benefit from structured educational courses to enhance their technology skill acquisition, as well as enable them to bridge their theoretical knowledge with workplace policies and expectations [17,39,43,44]. This also should include exposure to and education about the wide range of medical devices [45] used in the clinical setting (eg, infusion pumps, monitors, and parenteral nutrition devices), having access to consistent DHT support resources, particularly during night shifts and after hours, having opportunities for continuing education in digital health, and providing support for CNEs and nurses serving in clinical preceptor roles, and to all nurses according to their educational needs [4,11,17,39,40]. Collectively, this would improve nurses’ inclination, uptake, and competency in digital health technologies, as well as facilitate a healthy organizational culture that values innovation, change, and a growth mindset; hence, creating a win-win situation.

Furthermore, policies at the professional, organizational, and health system levels directly influence nursing practice in digital health and have implications for the quality of care, patient outcomes, health care service delivery, and quality improvement [1,8,9,38,39]. Currently, there is no digital health strategy for Canada and the eHealth strategy for nurses is outdated. Creating or updating these policies would help outline the digital health vision at the health system level, clarify the professional expectations for nurses according to their scope of practice, as well as other members of the health care team, facilitate the development of supporting and comprehensive digital health infrastructures including adequate funding, education, resource allocation, and strategies that promote innovation, creativity, continuous learning, and improvement, which ultimately facilitates optimal practice with DHT to improve patient and health care system outcomes [38-40]. More research is needed to explore how digital health knowledge gaps experienced by new graduates affect their entry to practice and transition to the workplace and their overall career trajectory. Interventional studies are also needed to evaluate new graduates’ actual use of digital health technologies in the workplace and the impact on patient and organizational outcomes. Future studies are also needed to examine the role of clinical preceptors and clinical educators in developing competency in digital health. Although prior research has explored the status of digital health integration across Canadian schools of nursing [20,21], more research is needed that compares curricula between nursing programs and possibly other health professions.

### Strengths and Limitations

To our knowledge, this is the first study involving newly graduated nurses and their practice with DHT in the workplace within the Canadian context involving participants from 2 provinces. In addition, the use of qualitative interviews enabled the collection of rich data about participants’ experiences. Given that the data collected in this study is unique to the settings included in the research, this should be considered in the interpretation of the results and the transferability of findings to other contexts.

### Conclusions

Although the study included participants from EC and WC, findings revealed more similarities rather than differences between participants with respect to digital health education, technology-related challenges, and their influence on nursing practice. Key strengths that newly qualified nurses in this study had included digital capabilities, awareness, and adaptability. However, inconsistent, or inadequate education about digital health during prelicensure nursing education, a proficiency-based approach to working with technology in the workplace, and variable technological infrastructure and support resources available created significant practice-related challenges that can detract nurses from patient care and limit optimal use of DHT. Digital health is the foundation of contemporary health care; therefore, comprehensive education about digital health during nursing school and throughout professional nursing practice, as well as organizational support and policy, are critical pillars. Health systems investing in DHT must also strive to create supportive work environments for nurses to thrive in technologically rich practice settings and further develop their capacity to deliver the digital health future.

## Appendix

Multimedia Appendix 1 Codes, categories and themes of interviews with new graduate nurses employed in the workplace.

## Abbreviations

AI: artificial intelligence
CIS: clinical information system
CNE: clinical nurse educator
DHT: digital health technology
EC: Eastern Canada
RA: research assistant
RN: registered nurse
WC: Western Canada

## References

[1] Spatharou A, Hieronimus S, Jenkins J (2020). Transforming healthcare with AI: the impact on the workforce and organizations: executive briefing.

[2] Snowdon A Digital health: a framework for healthcare transformation white paper. HIMSS.

[3] Brown TMH, Bewick M (2023). Digital health education: the need for a digitally ready workforce. Arch Dis Child Educ Pract Ed.

[4] Wong BLH, Khurana MP, Smith RD, El-Omrani O, Pold A, Lotfi A, O'Leary CA, Saminarsih DS (2021). Harnessing the digital potential of the next generation of health professionals. Hum Resour Health.

[5] McMillan K, Akoo C, Catigbe-Cates A (2023). New graduate nurses navigating entry to practice in the COVID-19 pandemic. Can J Nurs Res.

[6] Fatehi F, Samadbeik M, Kazemi A (2020). What is digital health? Review of definitions. Stud Health Technol Inform.

[7] (2021). Global strategy on digital health 2020-2025.

[8] (2023). Digital health transformation and nursing practice. International Council of Nurses (ICN).

[9] Government of Canada (2024). Nursing retention toolkit: improving the working lives of nurses in Canada.

[10] Canadian Association of Schools of Nursing (2012). Nursing informatics entry-to-practice competencies for registered nurses.

[11] Kleib M, Nagle L, Furlong K, Paul P, Wisnesky UD, Ali S (2022). Are future nurses ready for digital health?: Informatics competency baseline assessment. Nurse Educ.

[12] Kleib M, Jackman D, Wisnesky UD, Ali S (2021). Academic electronic health records in undergraduate nursing education: mixed methods pilot study. JMIR Nurs.

[13] Ewertsson M, Bagga-Gupta S, Allvin R, Blomberg K (2017). Tensions in learning professional identities—nursing students' narratives and participation in practical skills during their clinical practice: an ethnographic study. BMC Nurs.

[14] Kleib M, Nagle L (2018). Development of the Canadian nurse informatics competency assessment scale and evaluation of Alberta's registered nurses' self-perceived informatics competencies. Comput Inform Nurs.

[15] Kleib M, Nagle L (2018). Factors associated with Canadian nurses' informatics competency. Comput Inform Nurs.

[16] van Houwelingen CTM, Ettema RGA, Kort HSM, Cate OT (2017). Internet-generation nursing students' view of technology-based health care. J Nurs Educ.

[17] Brown J, Pope N, Bosco AM, Mason J, Morgan A (2020). Issues affecting nurses' capability to use digital technology at work: an integrative review. J Clin Nurs.

[18] Raghunathan K, McKenna L, Peddle M (2023). Baseline evaluation of nursing students' informatics competency for digital health practice: a descriptive exploratory study. Digit Health.

[19] Warshawski S (2020). Israeli nursing students' acceptance of information and communication technologies in clinical placements. J Prof Nurs.

[20] Nagle L, Kleib M, Furlong K (2020). Digital health in Canadian schools of nursing part aducators' perspectives. Qual Adv Nurs Educ.

[21] Nagle L, Kleib M, Furlong K (2020). Digital health in Canadian schools of nursing part B: academic administrators' perspectives. Qual Adv Nurs Educ.

[22] Mollart L, Newell R, Noble D, Geale SK, Norton C, O'Brien AP (2021). Nursing undergraduates’ perception of preparedness using patient electronic medical records in clinical practice. AJAN.

[23] Shin EH, Cummings E, Ford K (2018). A qualitative study of new graduates' readiness to use nursing informatics in acute care settings: clinical nurse educators' perspectives. Contemp Nurse.

[24] Caton E, Philippou J, Baker E, Lee G (2024). Exploring perceptions of digital technology and digital skills among newly registered nurses and clinical managers. Nurs Manag (Harrow).

[25] Doyle L, McCabe C, Keogh B, Brady A, McCann M (2020). An overview of the qualitative descriptive design within nursing research. J Res Nurs.

[26] DeJonckheere M, Vaughn LM (2019). Semistructured interviewing in primary care research: a balance of relationship and rigour. Fam Med Community Health.

[27] Malterud K, Siersma VD, Guassora AD (2016). Sample size in qualitative interview studies: guided by information power. Qual Health Res.

[28] Bengtsson M (2016). How to plan and perform a qualitative study using content analysis. NursingPlus Open.

[29] Elo S, Kyngäs H (2008). The qualitative content analysis process. J Adv Nurs.

[30] Vaismoradi M, Turunen H, Bondas T (2013). Content analysis and thematic analysis: implications for conducting a qualitative descriptive study. Nurs Health Sci.

[31] Bove LA, Sauer P (2023). Nursing faculty informatics competencies. Comput Inform Nurs.

[32] Chauvette A, Kleib M, Paul P (2022). Developing nursing students' informatics competencies—a Canadian faculty perspective. Int J Nurs Educ Scholarsh.

[33] Kleib M, Chauvette A, Furlong K, Nagle L, Slater L, McCloskey R (2021). Approaches for defining and assessing nursing informatics competencies: a scoping review. JBI Evid Synth.

[34] Forman TM, Armor DA, Miller AS (2020). A review of clinical informatics competencies in nursing to inform best practices in education and nurse faculty development. Nurs Educ Perspect.

[35] Nazeha N, Pavagadhi D, Kyaw BM, Car J, Jimenez G, Tudor Car L (2020). A digitally competent health workforce: scoping review of educational frameworks. J Med Internet Res.

[36] Booth RG, Strudwick G, McBride S, O’Connor S, López ALS (2021). How the nursing profession should adapt for a digital future. BMJ.

[37] Booth RG, Strudwick G (2021). Preparing nursing for the virtual care realities of a post-pandemic future. Nurs Leadersh (Tor Ont).

[38] Troncoso EL, Breads J (2021). Best of both worlds: digital health and nursing together for healthier communities. Int Nurs Rev.

[39] (2024). Clinical practice in a digital health environment. RNAO.

[40] Subramanian S, Kleib M (2023). Leveraging clinical preceptorship to enhance nursing students’ readiness in digital health. Qual Adv Nurs Educ.

[41] Wilson CB, Slade C, Wong WYA, Peacock A (2020). Health care students experience of using digital technology in patient care: a scoping review of the literature. Nurse Educ Today.

[42] Edirippulige S, Samanta M, Armfield NR (2018). Assessment of self-perceived knowledge in e-Health among undergraduate students. Telemed J E Health.

[43] Lindfors K, Kaunonen M, Huhtala H, Paavilainen E (2022). Newly graduated nurses' evaluation of the received orientation and their perceptions of the clinical environment: an intervention study. Scand J Caring Sci.

[44] McGarity T, Monahan L, Acker K, Pollock W (2023). Nursing graduates' preparedness for practice: substantiating the call for competency-evaluated nursing education. Behav Sci (Basel).

[45] Ewertsson M, Gustafsson M, Blomberg K, Holmström IK, Allvin R (2015). Use of technical skills and medical devices among new registered nurses: a questionnaire study. Nurse Educ Today.

