# Supplementary material for: Newly Qualified Canadian Nurses’ Experiences With Digital Health in the Workplace: Comparative Qualitative Analysis
Source: JMIR Med Educ. 2024 Aug 19;10:e53258. doi: 10.2196/53258 (PMC11369539; doi:10.2196/53258)
Supplement: Multimedia Appendix 1 [file mededu_v10i1e53258_app1.docx]

| **Code** | | **Category** | **Themes** |
| --- | --- | --- | --- |
| - Limited and broad education about DH; No hands-on experiences in lab. - Theory and practice gap. | | Academic | **Experiences prior to becoming an RN.** |
| - Learning about multiple clinical information systems (old and new). - Variable learning opportunities about electronic charting. - Being tech savvy takes away opportunities for learning about DH. | | Clinical |  |
| - Use of technology in personal life enhances self-efficacy. | | Personal experiences |  |
| - Assists with prioritization of care. - Facilitates continuity of timely care. | | Technology is an essential tool for nurses’ work | **Experiences Upon Joining the Work Environment.** |
| - Different charting techniques and proficiency among nurses. - Learning curve and experience level. | | Adaptability to overcome the learning curve |  |
| - Learning from and teaching other nurses. - Resistance from senior nurses to DH technologies. - Role models and supportive unit culture. | | Learning about DHT is a social process |  |
| - Different unit charting practices can be confusing. - Potential legal liabilities. | | Unclear or violation of org. policies and procedures |  |
| - Disconnected and hybrid and old systems. - Not enough computer stations and slow roll out of DH technologies. | | IT infrastructure challenges |  |
| - Using different systems to do my work (Medical devices; Hardware; CIS) - Accuracy problems with older devices. - IT glitches are not resolved quickly. - No technical support during night shifts. - Variable IT support | | IT-related practice challenges |  |
| - Access to learning is available only during day shift. - On the job-training on CIS; No formal training on use of medical devices. - Playground or sandbox during training. - Basic training upon orientation — learn as you go. - Training - condensed training over 2 days. - Training across the organization—wider support network to new grads. | | Variable Training (Clinical information systems (CIS) & medical devices) |  |
| - Dedicated courses about DH and core topics. - Early exposure to DH technology to enhance confidence and competence. | | Nursing education strategies | **Strategies for Bridging the Gap in Transition to DH Practice.** |
| People-related   - - Leadership and advocacy.   - New hires need more time and ongoing support.   - End user perspectives. | Technology-related   - - Education and support about medical devices.   - More integrated DH technologies in all healthcare settings. | Work setting strategies |  |
